# Association of weight change following smoking cessation with the risk of tuberculosis development: A nationwide population-based cohort study

**DOI:** 10.1371/journal.pone.0266262

**Published:** 2022-04-07

**Authors:** Seung Hoon Kim, Yong-Moon Park, Kyungdo Han, Seung Hyun Ko, Shin Young Kim, So Hyang Song, Chi Hong Kim, Kyu Yeon Hur, Sung Kyoung Kim

**Affiliations:** 1 Division of Pulmonology, Department of Internal Medicine, St. Vincent’s Hospital, College of Medicine, The Catholic University of Korea, Suwon, Korea; 2 Department of Epidemiology, Fay W. Boozman College of Public Health, University of Arkansas for Medical Sciences, Little Rock, Arkansas, United States of America; 3 Department of Statistics and Actuarial Science, Soongsil University, Seoul, Korea; 4 Division of Endocrinology and Metabolism, Department of Internal Medicine, St. Vincent’s Hospital, College of Medicine, The Catholic University of Korea, Suwon, Korea; 5 Division of Endocrinology and Metabolism, Department of Medicine, Samsung Medical Center, Sungkyunkwan University School of Medicine, Seoul, Korea; Osakidetza Basque Health Service, SPAIN

## Abstract

**Background:**

Smoking or weight loss is a risk of tuberculosis (TB) development. However, the impact of weight change after smoking cessation on the occurrence of TB remains elusive. We aimed to determine the relationship between weight change after smoking cessation and the risk of TB development.

**Methods:**

We conducted a population-based cohort study using the national database in Republic of Korea. Of the 10,490,491 subjects who underwent health check-up in 2009, we enrolled 9,953,124 subjects without a previous TB history and followed them until 2017. We divided all study participants into the following three groups: never, former, and current smokers. The primary endpoint was newly developed TB.

**Results:**

Among 9,953,124 subjects analyzed, 5,922,845 (59.5%) were never smokers, 1,428,209 (14.4%) were former smokers, and 2,602,080 (26.1%) were current smokers. The risk of TB development was significantly higher in current smokers than in never smokers (adjusted hazard ratio (aHR) 1.158; 95% confidence interval [CI] 1.131–1.186). Among current smokers, individuals who stopped smoking and maintained weight after baseline evaluation had a significantly lower risk of TB development compared with those who continued to smoke (aHR 0.771; 95% CI 0.741–0.892). However, even after smoking cessation, individuals who lost weight were at a significantly higher risk of TB development compared with those who continued to smoke (aHR 1.327; 95% CI 1.119–1.715).

**Conclusions:**

Our findings suggest that smoking is a risk factor for TB and weight maintenance (neither gaining or losing) after quitting smoking might reduce the risk of TB development.

## Introduction

Tuberculosis (TB) is a respiratory transmission disease caused by the Mycobacterium tuberculosis. It is one of the highly infectious diseases, which infects about 30% of those who have had close contact with it. About 10% of infected people develop to active TB over their lifetime [1]. In addition, this disease carries a high worldwide burden, with more than 10 million new cases and 1.2 million deaths annually [2]. Accordingly, the United Nations (UN) has included a TB management strategy in its Sustainable Development Goals (SDGs) following the Millennium Development Goals (MDGs) [3, 4]. The World Health Organization (WHO) is making efforts to reduce TB incidence and death worldwide through its END TB strategy, following its STOP TB strategy [5, 6]. According to the WHO report, the incidence and mortality rates are decreasing as compared to the past in Korea [2]. But as of 2018, about 34,000 people were diagnosed with TB, resulting in an incidence rate of 66 per 100,000 people, which is still high compared to other OECD countries [2]. There are still 4.8 deaths per 100,000 people in all OECD countries [2]. Because of the high incidence and mortality rate of TB, multilateral TB management projects are required [2].

Smoking is one of the most important preventable causes of premature death in the world. More than 6 million people per year die from smoking across the globe [7]. There is no question that limiting smoking is one of the most effective ways to save lives and improve overall well-being. In 2016, the WHO reported that there are 1.3 billion smokers, or about 20% of the world’s population [7]. The breakdown by sex shows that 33.7% are men and 6.2% are women [7]. Smoking is known to increase the risk of pulmonary TB and to affect TB recurrence and treatment delay [8, 9]. Also, smoking results in treatment failure, delayed negative conversion of sputum smear, disease exacerbation, drug-resistant TB, and it increases mortality during and after treatment [8, 9]. Thus, smoking cessation is very important in improving treatment outcomes and lowering mortality among TB patients [10, 11].

As mentioned above, quitting smoking is critical for the prevention and management of TB and other diseases, on the other hand, it can also cause various physiological changes in the body [12]. One of them is weight change. Some studies reported weight gain after quitting smoking [13, 14], but another study reported weight loss after quitting smoking [15]. In general, most studies have concluded that the relationship between weight change and TB is that weight loss is a risk factor for TB and that overweight has a protective effect against TB [16, 17]. However, no research has been conducted on how weight changes following smoking cessation affects the development of TB.

Therefore, the primary objective of this study was to investigate the impact of smoking on the occurrence of TB in healthy people without a history of TB, and the secondary objective was to determine how the weight change following smoking cessation in these people affects the development of TB using National Health Insurance Service (NHIS) database in Korea.

## Materials and methods

### Data source and study population

**Fig 1:**
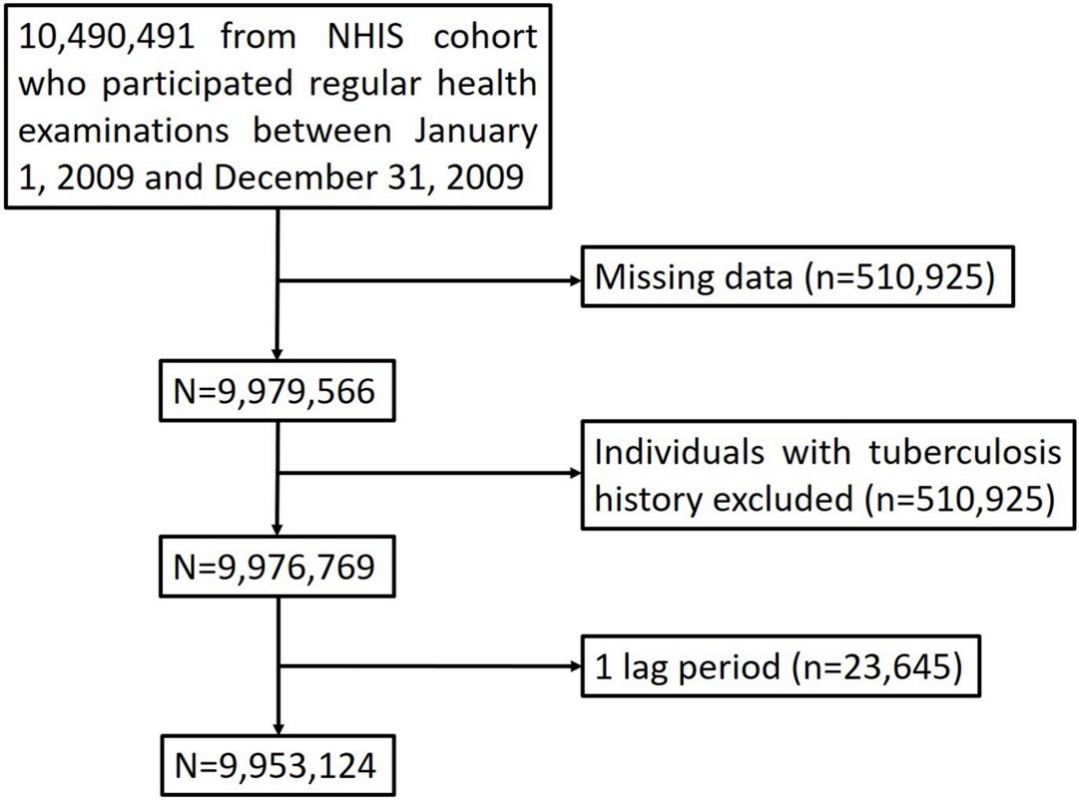
Flow chart of the study population. 9,943,124 participants were enrolled in this study. They were followed up until December 31, 2017. NHIS, national health insurance service.

In the Republic of Korea, 97% of all nationals (about 51,000,000 people) are enrolled in the NHIS, a mandatory health insurance program, at the national level [18]. The NHIS collects all medical records of all nationals registered in this health insurance system. This NHIS database contains subscriber demographics, inpatient and outpatient medical use, and claim data for prescribed medications and performed procedures. In addition, NHIS recommends a regular health examination once every two years for all insured adults aged 20 years and older. Health examination items include body measurements such as height and weight, as well as blood pressure and blood tests. In addition, through the questionnaire survey, items such as past medical history, family history, smoking and drinking history, and physical activity are investigated. Since 2015, the NHIS database has been opened to all researchers whose study protocols are approved by an official review committee. Using this NHIS database, we conducted a population-based retrospective cohort study. Eligible participants were adults aged 20 years and older who had undergone a regular health examination between January 1, 2009 and December 31, 2009 (n = 10,490,491). Among them, those with a history of TB before January 1, 2009and those with any missing data were excluded. Finally, 9,953,124 subjects were enrolled and analyzed (Fig 1). We followed the subjects from January 1, 2009 to December 31, 2017. A median follow-up period was 7.3 (7.1–7.6) years. This study was approved by the Institutional Review Board (IRB) of St. Vincent’s Hospital, The Catholic University of Korea (IRB number: VC20ZISI0014) and the NHIS (research number: REQ0000035808). Informed consent was waived by the board.

### Definitions

We evaluated data on demographics and health examination items. We classified smoking status based on the self-reported questionnaire records as follows: We defined current smokers as those who had smoked more than five packs or a total of 100 cigarettes throughout their lifetime and who continued to smoke. We defined former smokers as those who had smoked more than five packs or a total of 100 cigarettes throughout their lifetime but who had quit smoking. We defined never smokers as those who had smoked five packs or fewer [19, 20]. Both former and current smokers recorded the number of years they had smoked and the average daily number of cigarettes they had smoked into the self-report questionnaire. We reported the cumulative lifetime smoking exposure in terms of the pack-year, derived by multiplying the average number of cigarette packs consumed per day times the number of years they had smoked. We identified heavy drinkers as being those individuals who consumed more than 30 g of alcohol per day on average [21, 22]. We also defined regular exercise as being intense physical activity that made breathing faster than usual on more than 3 days a week for at least 20 min at a time, or as being moderate physical activity that made breathing slighter faster than usual on more than five days a week for at least 30 min at a time [21, 22].

We also identified diabetes mellitus (DM), hypertension, and dyslipidemia as baseline comorbidities in the study population, using the International Classification of Disease, Tenth Revision (ICD-10) codes and additional information, as in a previous work [21, 22]. We defined participants as having DM if they had a fasting blood glucose level of 126 mg/dL or greater, or if they had been diagnosed by using ICD-10 codes E11-14, as well as having been prescribed medications for DM. We defined participants as having hypertension if they had a systolic blood pressure of 140 mmHg or greater, a diastolic blood pressure of 90 mmHg or greater, or if they had been diagnosed by using ICD-10 codes I10-13 and I15, as well as having been prescribed antihypertensive medications [21, 22]. We defined participants as having dyslipidemia if they had a fasting serum total cholesterol of 240 mg/dL or more or they had been diagnosed by using the ICD-10 code E78, as well as having been prescribed lipid-lowering agents [21, 22]. We defined participants as having any cancer if they had been diagnosed by using the ICD-10 C code. A history of cerebrovascular or heart disease was defined through self-reported questionnaires at the point of the examination. Among current smokers, subject who underwent a regular health examination within 2 years after baseline were classified into quit-smoking and continued-smoking group according to the smoking status at the regular health examination. For example, subjects who were current smoker at baseline but quit before the regular health examination were classified into the quit-smoking group, and those who current smoker at both baseline and regular health examination were classified into the continued-smoking group. We additionally divided each of these into weight-loss, weight-maintenance, and weight-gain group, depending upon whether they had had a 5% or greater weight change at their regular health examination after two years compared to their baseline [23, 24].

### Study outcome

The primary outcome was newly diagnosed TB during the follow-up period. Since 2005, the Korean government has implemented a policy to expand NHIS benefit coverage to provide financial protection against catastrophic diseases such as cancer and TB. This NHIS program reimburses 80% to 100% of the costs of catastrophic diseases such as TB. When patients with TB are registered in this system, they are assigned a special code (V code; V206 or V246). We identified patients with TB by using both ICD-10 (A15-A19) and V codes, as had been done in a previous study [21].

### Statistical analysis

We expressed data both as the mean ± standard deviations for continuous variables and as proportions for categorical variables. We used the Student’s *t* test and analysis of variance to compare the differences between continuous variables, and we used the chi-square test to compare the differences between categorical variables. The incidence rates of TB were calculated and expressed as the number of events per 1,000 person-years. We compared the cumulative incidences of TB between groups by using the Kaplan-Meier method and the log-rank test. We used the multivariable Cox proportional hazards models to analyze the adjusted risk of TB development, based on smoking status and the daily amount or duration of smoking, and we described the results as hazard ratios (HR) with 95% confidence intervals (CI). Potential confounders were identified as a priority based on literature review. The following baseline covariates were included in multivariable adjusted models: age and sex for model 1; age, sex, alcohol consumption status, regular exercise, income level, BMI, and cancer for model 2. We considered a *p* value of less than 0.05 to be statistically significant. We performed the statistical analyses by using SAS version 9.4 (SAS Institute, Cary, NC, USA).

## Results

### Baseline characteristics of the study populations

Of the 10,490,491 subjects who underwent regular health examinations between January 1, 2009 and December 31, 2009, 9,953,124 subjects were enrolled and analyzed (Fig 1). Among 9,953,124 subjects analyzed, 5,922,845 (59.5%) were never smokers, 1,428,209 (14.4%) were former smokers, and 2,602,080 (26.1%) were current smokers. Former and never smokers were significantly older than current smokers (*p* <0.0001). Male sex and heavy drinking were significantly associated with former or current smoking (*p* <0.0001). Study subjects exercising regularly were significantly more likely to be former or current smokers than were the never smokers (*p* <0.0001). The former smokers were significantly more obese than the never or current smokers (*p* <0.0001). Compared with the never and current smokers, former smokers had significantly higher blood pressure, fasting blood glucose, and serum total cholesterol levels, as well as a higher prevalence of DM, hypertension, and dyslipidemia (*p* <0.0001) (Table 1).

**Table 1:** Baseline characteristics of the study population.

	Never smoker	Former smoker	Current smoker
	(n = 5,922,835)	(n = 1,428,209)	(n = 2,602,080)
Age, years	48.6±14.6	48.9±13.1	42.7±12.5
Age, years			
20–39	1,580,687 (26.7)	365,168 (25.6)	1,173,647 (45.1)
40–64	3,402,213 (57.4)	870,002 (60.9)	1,259,612 (48.4)
≥65	939,935 (15.9)	193,039 (13.5)	168,821 (6.5)
Male sex	1,659,315 (28.0)	1,343,846 (94.0)	2,447,270 (94.0)
Height, cm	160±8.5	169±6.7	170±7.0
Weight, kg	60.2±10.5	69.6±10.2	69.2±11.3
Waist circumference, cm	78.3±9.2	83.9±7.9	82.6±8.2
BMI, kg/m^2^	23.5±3.3	24.3±2.9	23.9±3.2
BMI, kg/m^2^ (grade)			
<18.5	264,922 (4.5)	26,201 (1.8)	85,796 (3.3)
18.5–23	2,504,855 (42.3)	439,854 (30.8)	971,779 (37.4)
23–25	1,410,700 (23.8)	406,272 (28.5)	648,682 (24.9)
25–29	1,546,411 (26.1)	509,339 (35.7)	794,195 (30.5)
≥30	195,947 (3.3)	46,543 (3.3)	101,628 (3.9)
Systolic blood pressure, mmHg	121.4±15.4	125±14.2	123.4±13.9
Diastolic blood pressure, mmHg	75.4±10.0	78.2±9.7	77.4±9.7
Fasting glucose, mg/dL	96.1±21.5	100.1±24.2	97.8±24.8
Hemoglobin, g/dL	13.3±1.5	14.7±1.3	15±1.3
Total cholesterol, mg/dL	195±36.8	196±36.1	194.6±36.4
Triglyceride ^a^, mg/dL	102.6	126.3	135.7
	(102.5–102.6)	(126.2–126.4)	(135.6–135.8)
Rural residence	3,177,187 (53.6)	753,403 (52.8)	1,440,503 (55.4)
Heavy drinker ^b^	118,322 (2.0)	162,953 (11.4)	400,242 (15.4)
Regular exercise ^c^	2,755,052 (46.5)	922,273 (64.6)	1,441,620 (55.4)
Comorbidity			
Diabetes	482,420 (8.2)	159,253 (11.2)	223,992 (8.6)
Hypertension	1,544,220 (26.1)	451,319 (31.6)	564,536 (21.7)
Dyslipidemia	1,126,252 (19.0)	287,475 (20.1)	405,518 (15.6)
Cerebrovascular disease	52,141 (1.4)	26,652 (2.8)	23,088 (0.9)
Heart disease	115,569 (3.1)	46,519 (4.8)	36,346 (1.4)

BMI, body mass index. Values are number (%) or mean ± standard deviation.

^a^ Geographic means.

^b^ Defined as a person who drinks more than 30 gram of alcohol a day on average.

^c^ Defined as high-density exercise on more than 3 days a week for at least 20 minutes at a time or moderate-intensity exercise on more than 5 days a week for at least 30 minutes at a time.

### Incidence and risk of TB according to smoking status

**Fig 2:**
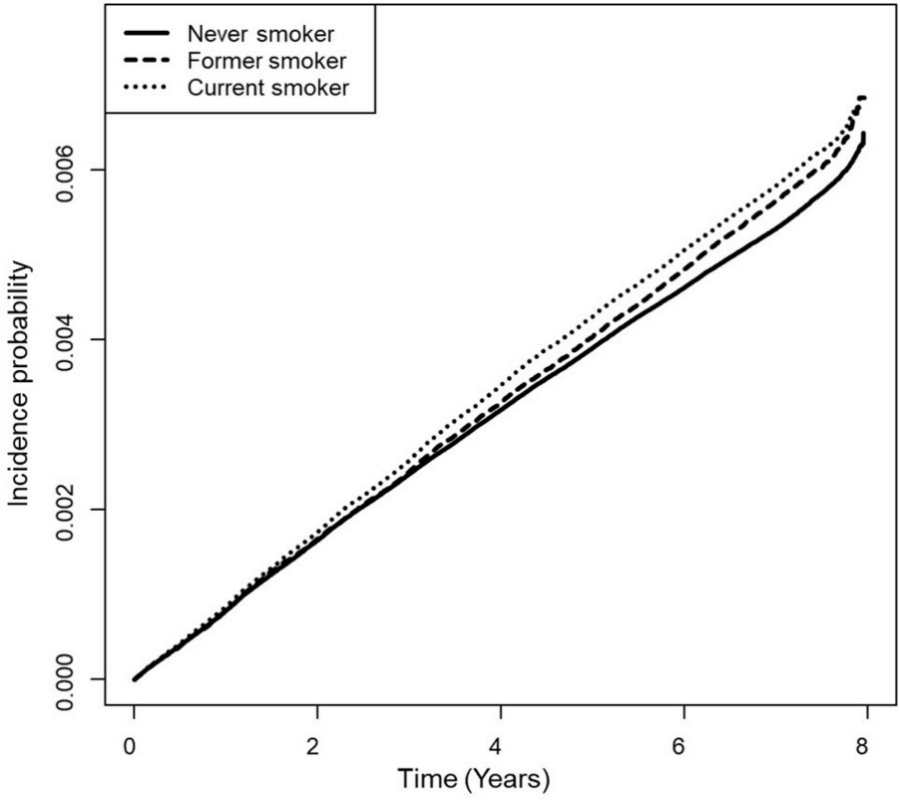
Cumulative incidence curve of tuberculosis according to smoking status by Kaplan-Meier method and log-rank test (*P* < 0.0001).

After a median follow-up period of 7.3 (7.1–7.6) years, TB occurred in 32,966 never smokers (0.56%), 8,365 former smokers (0.59%), and 15,664 (0.60%) current smokers (Table 2). The cumulative incidence of TB in the current smokers had increased at a constant rate following baseline, and it was significantly higher than it had been in the former and never smokers (*p* <0.0001) (Fig 2). The risk of TB development after adjusting for potential confounders was significantly higher in the current smokers (aHR 1.158; 95% CI 1.130–1.186) but lower in the former smokers (aHR 0.947; 95% CI 0.921–0.973) compared to the never smokers (model 2 in Table 2).

**Table 2:** Incidence and risk of tuberculosis according to smoking status.

Smoking status	Total no. (n)	TB cases (n)	TB incidence	HR (95% CI)	
			(per 1,000 person-years)	Model 1 ^a^	Model 2 ^b^
Never smokers	5,922,835	32,966	0.77	1 (reference)	1 (reference)
Former smokers	1,428,209	8,365	0.81	0.906 (0.881–0.932)	0.947 (0.921–0.973)
Current smokers	2,602,080	15,664	0.83	1.257 (1.228–1.287)	1.158 (1.130–1.186)

TB, tuberculosis; HR, hazard ratio; CI, confidential interval.

^a^ Model 1: adjusted for age and sex.

^b^ Model 2: adjusted for model 1 + alcohol consumption status, regular exercise, income level, and body mass index.

### Risk of TB according to the amounts and durations of cigarettes smoked

**Fig 3:**
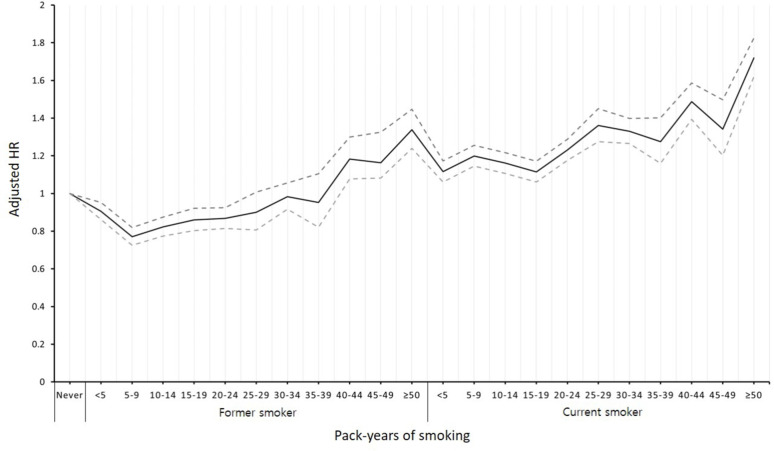
Risk of tuberculosis development according to the cumulative amounts of cigarettes smoked. Hazard ratios were adjusted for age, sex, alcohol consumption status, regular exercise, income level, and body mass index. Solid line indicates adjusted hazard ratios and dashed lines indicate 95% confidence interval, respectively. HR, hazard ratio.

We evaluated the risk of TB development according to the daily amounts of cigarettes smoked in the former- and current-smoker groups (Table 3). Compared to never-smoker group, the risk of TB development, based on the average cigarette consumption per day, showed a dose-response relationship in the current-smoker group (aHR, 1.061 [95% CI 1.004–1.057] in individuals who smoked less than 0.5 pack per day; aHR, 1.097 [95% CI 1.062–1.132] in individuals who smoked 0.5~1 pack per day; aHR, 1.267 [95% CI 1.230–1.304] in individuals who smoked 1 or more pack per day) (*p* for trend <0.0001). In former smokers, the risk of TB development was significantly lower compared to the never smokers (aHR, 0.918 [95% CI 0.869–0.971] in individuals who smoked fewer than 0.5 pack per day; aHR, 0.885 [95% CI 0.850–0.923] in individuals who smoked 0.5~1 pack per day). However, we did not identify this inverse association in former smokers who had smoked more than one pack per day (model 2 in Table 3). There was a significant dose-response relationship between the risk of TB development and how long the current smokers had smoked (Table 4). Compared to the never smokers, the aHRs of TB development among the current smokers were 1.154 (95% CI 1.120–1.189), 1.228 (95% CI 1.164–1.295), and 1.369 (95% CI 1.329–1.411) in individuals who had smoked cigarettes for fewer than 10, 10~29, and 30 years or more, respectively (*p* for trend <0.0001). Former smokers who had smoked cigarettes for fewer than 30 years showed a decreased risk of TB development compared to the never smokers. However, former smokers who had smoked cigarettes for over 30 years showed an increased risk of TB (aHR, 1.137 [95% CI 1.091–1.186] in model 2). Based on the effect of cumulative lifetime smoking exposure on TB development, current smokers showed a dose-response relationship in the incidence of TB in all sections of smoking amounts. However, the incidence of tuberculosis of former smokers had a dose-response relationship only in groups over 40 pack-years in model 2 (Fig 3).

**Table 3:** Incidence and risk of tuberculosis according to daily smoking amount.

Smoking status	No. of cigarettes smoked per day	Total no. (n)	TB cases (n)	TB incidence	HR (95% CI)	
				(per 1,000 person-years)	Model 1 ^a^	Model 2 ^b^
Never smokers	-	5,922,835	32,966	0.77	1 (reference)	1 (reference)
Former smokers	<10	255,707	1,351	0.73	0.923 (0.873–0.976)	0.918 (0.869–0.971)
	10–19	543,177	2,798	0.7	0.857 (0.822–0.893)	0.885 (0.850–0.923)
	≥20	629,325	4,216	0.9	0.944 (0.911–0.977)	1.015 (0.980–1.051)
Current smokers	<10	325,763	1,801	0.77	1.119 (1.066–1.175)	1.061 (1.004–1.057)
	10–19	1,093,581	5,750	0.73	1.195 (1.158–1.234)	1.097 (1.062–1.132)
	≥20	1,182,736	8,113	0.95	1.352 (1.314–1.391)	1.267 (1.230–1.304)

TB, tuberculosis; HR, hazard ratio; CI, confidential interval.

^a^ Model 1: adjusted for age and sex.

^b^ Model 2: adjuster for model 1 + alcohol consumption status, regular exercise, income level, and body mass index.

*p* for trend < 0.0001 for HR in former and current smokers.

**Table 4:** Incidence and risk of tuberculosis according to smoking duration.

Smoking status	Duration of smoking (years)	Total no. (n)	TB cases (n)	TB incidence	HR (95% C.I)	
				(per 1,000 person-years)	Model 1 ^a^	Model 2 ^b^
Never smokers	-	5,937,726	33,042	0.77	1 (reference)	1 (reference)
Former smokers	<10	361,780	1,448	0.55	0.718 (0.681–0.756)	0.899 (0.852–0.949)
	10–29	832,266	4,082	0.68	0.884 (0.855–0.913)	0.799 (0.771–0.828)
	≥30	227,980	2,804	1.77	2.302 (2.215–2.392)	1.137 (1.091–1.186)
Current smokers	<10	418,853	1,560	0.51	1.668 (1.635–1.703)	1.154 (1.120–1.189)
	10–29	1,634,772	7,418	0.63	1.818 (1.797–1.838)	1.228 (1.164–1.295)
	≥30	539,747	6,641	1.76	2.297 (2.237–2.358)	1.369 (1.329–1.411)

TB, tuberculosis; HR, hazard ratio; CI, confidential interval.

^a^ Model 1: adjusted for age and sex.

^b^ Model 2: adjuster for model 1 + alcohol consumption status, regular exercise, income level, and body mass index.

*p* for trend < 0.0001 for HR in former and current smokers.

### Association of smoking cessation and weight change with the risk of TB

Of the 2,602,080 current smokers, 1,533,250 (58.9%) had their regular health examinations within two years, consecutively. Of these, 215,479 (14.1%) had quit smoking before their regular health examinations (quit-smoking group) and 1,317,711 (85.9%) had continued smoking (continued-smoking group). We divided each of these into weight-loss, weight-maintenance, and weight-gain group, based on a 5% or greater weight change noted on their regular health examination compared to their baseline (Table 5). In the quit-smoking group, weight loss, weight maintenance, and weight gain were 14,389 (6.7%), 141,366 (65.6%), and 59,724 (27.7%), respectively, and in the continued-smoking group, 121,116 (9.2%), 986,765 (74.9%), and 209,890 (15.9%), respectively. When the risk of TB in the continued-smoking and weight-maintenance group is set to 1, the risk of TB development in the continued-smoking and the weight-loss group and quit-smoking and weight-loss group is 1.401 (95% CI 1.224–1.518) and 1.389 (95% CI 1.158–1.712). However, the risk of TB development in the quit-smoking and weight-maintenance group was 0.811 (95% CI 0.791–0.916), which was significantly lower than in the continued-smoking and weight-maintenance group, the continued-smoking and weight-loss group, or the quit-smoking and weight-loss group. Also, the risk of TB development in the quit-smoking and weight-maintenance group was significantly lower than in the all continued-smoking group regardless of the weight changes (aHR 0.771; 95% CI 0.741–0.892) (Table 6). In the quit-smoking and weight-maintenance group, the proportion of heavy alcohol drinkers was significantly lower and the proportion of regular exercise was significantly higher than other groups (S1 Table).

**Table 5:** Incidence and risk of tuberculosis according to changes in smoking status and body weight among current smokers.

Current smoker’s smoking status within 2 years	Weight change	Total no. (n)	TB cases (n)	TB incidence	HR (95% C.I)	
				(per 1,000 person-years)	Model 1 ^a^	Model 2 ^b^
Continued smoking	Loss	121,116	745	1.18	1.427 (1.319–1.544)	1.401 (1.224–1.518)
	Maintenance	986,765	3,799	0.73	1 (reference)	1 (reference)
	Gain	209,890	644	0.58	0.980 (0.901–1.066)	0.951 (0.871–1.418)
Quit smoking	Loss	14,389	88	1.17	1.325 (1.072–1.637)	1.389 (1.158–1.712)
	Maintenance	141,366	481	0.64	0.775 (0.705–0.853)	0.811 (0.791–0.916)
	Gain	59,724	215	0.68	0.937 (0.817–1.075)	0.971 (0.855–1.143)

TB, tuberculosis; HR, hazard ratio; CI, confidential interval.

^a^ Model 1: adjusted for age and sex.

^b^ Model 2: adjusted for model 1 + alcohol consumption status, regular exercise, income level, and cancer.

**Table 6:** Incidence and risk of tuberculosis in quit-smoking group according to weight changes compared with all continued-smoking group regardless of weight changes.

Current smoker’s smoking status within 2 years	Weight change	Total no. (n)	TB cases (n)	TB incidence	HR (95% C.I)	
				(per 1,000 person-years)	Model 1 ^a^	Model 2 ^b^
Continued smoking	All	1,317,771	5188	0.74	1 (reference)	1 (reference)
Quit smoking	Loss	14,389	88	1.17	1.265 (1.025–1.563)	1.327 (1.119–1.715)
	Maintenance	141,366	481	0.64	0.742 (0.676–0.815)	0.771 (0.741–0.892)
	Gain	59,724	215	0.68	0.898 (0.784–1.030)	0.949 (0.847–1.173)

TB, tuberculosis; HR, hazard ratio; CI, confidential interval.

^a^ Model 1: adjusted for age and sex.

^b^ Model 2: adjusted for model 1 + alcohol consumption status, regular exercise, income level, and cancer.

## Discussion

In this nationwide population-based cohort study, we found that the risk of TB development was higher in the current smokers than in the never smokers, and the risk tended to increase with increasing amount and duration of smoking. In the former smokers, the risk of tuberculosis development was lower in those with smoking amount less than 20 pack-years or those with smoking duration less than 30 years compared with the never smokers. Among current smokers, individuals who stopped smoking and maintained weight after baseline evaluation had a significantly lower risk of TB development compared with than those who continued to smoke. However, even after smoking cessation, individuals who lost weight were at a significantly higher risk of TB development compared with those who continued to smoke. To our knowledge, this is the first study that shows weight maintenance (neither weight gain nor loss) after smoking cessation may reduce the risk of TB development in current smokers who have no history of TB.

This study followed a general population without a history of TB for nine years and analyzed cases of TB among them. As a result, it was confirmed that smoking is a risk factor for TB because of the high TB rate in the current-smoker group. In addition, it was shown that the incidence of TB increased proportionally with the duration and amount of smoking. Since Lowe reported that smoking may act as a predisposition to TB in 1956 [25], studies on the relationship between smoking and pulmonary TB have been conducted. To date, the conclusion from most previous studies is that smoking is a risk factor for the development of TB [26, 27]. In addition, treatment success rate for TB is significantly lower in the smokers than in the non-smokers [9]. Epidemiologically, about 1.3 billion people worldwide smoke, and most of them live in underdeveloped or developing countries with a high incidence or prevalence of TB [28]. Therefore, the biggest impact of smoking in terms of public health issues associated with infections may be to increase the risk of TB. Some systematic reviews and meta-analysis showed an unfavorable association between global epidemics of TB and smoking and exposure to smoking, active TB, and TB-related mortality associated with TB infection [26, 29].

When analyzing the relationship between TB incidence and weight change following smoking cessation in current smokers, the incidence of TB increased regardless of whether smoking continued or not as the weight decreased. This is consistent with most previous studies showing that low weight or BMI are associated with the development of TB [16, 17, 30, 31]. This can be explained as a poor immunity due to nutritional imbalance [32]. In other words, nutritional imbalance caused by underweight interferes with the immune system (eg, T cell suppression), which increases the risk of various infectious diseases, including tuberculosis. However, our study showed that the incidence of TB decreased in the groups who maintained weight (not weight gain or loss) after quitting smoking. These results of ours can be explained as follows. To quit smoking, in addition to simply quitting, lifestyle corrections are usually accompanied, such as abstinence or regular exercise [33]. In addition, it is known that smoking cessation sometimes leads to weight gain [13, 23, 34]. Therefore, current smokers are expected to do lifestyle modifications, such as exercise or diet control, along with quitting smoking to avoid weight gain following smoking cessation [35–37]. These lifestyle changes will have a positive effect on the management of chronic diseases, and even after attempting to quit smoking, the continued lifestyle corrections can lead to successful smoking cessation [38–40]. If lifestyle corrections accompany smoking cessation, various healthy body changes appear. Among them, proper weight maintenance is a representative example [41–43]. In other words, when current smokers quit smoking and maintain weight through lifestyle correction such as abstinence and regular exercise, this lifestyle correction naturally would lead to a healthy lifestyle pattern and contribute to the risk reduction of TB development. This relationship between smoking cessation, lifestyle correction, and weight maintenance can also be confirmed in our study. When we analyzed the basic characteristics of current smokers who had their regular health examinations after two years, we confirmed that the proportion of heavy alcohol drinkers was significantly lower and the proportion of regular exercisers was significantly higher in quit-smoking and weight-maintenance group than in other groups (S1 Table). That is, it has been shown that risk reduction of TB is possible even if weight management is made by steady lifestyle corrections in addition to quitting smoking.

Our study showed that the occurrence of TB in some groups of former smokers was less than in never smokers. These groups had a relatively low amount of smoking and a short smoking duration. This could imply that quitting smoking as soon as possible might be more helpful in achieving the risk reduction of TB.

Weight gaining after smoking cessation can be a potential barrier for quitting smoking and for maintenance abstinence, respectively. Professional help should encourage subjects to quit smoking at the same time as recommending a healthy lifestyle (proper physical activity or nutrition), but it is very important to encourage successful quitting subjects to continue abstinence, even if they gain weight [14, 44, 45].

Because our research is a retrospective study analyzing administrative data consisted of limited information, there are several inevitable limitations. First, since there was no information about the exact time when smokers quit smoking, the time-to-effect of smoking cessation on TB among smokers could not be fully evaluated. Also, the definitions of smoking status were made based on a questionnaire that only relied on the memory of the subjects. So, the potential for misclassification of smoking status would be possible. Second, because information regarding long-term follow-up data, including disease severity or mortality of the incident TB, was not also available from the NHIS database, the effect of smoking behavior on the severity or mortality of TB could also not be evaluated. Third, the analysis of the occurrence of TB according to smoking behavior and weight change was conducted only in the subgroup, not the entire population. However, this is also the result of data analysis at the level of one million people. Fourth, the effect of female sex on TB may have been underestimated because of the small proportion of current smokers who are women.

## Conclusions

In conclusion, our study showed that smoking is a risk factor for TB and maintaining weight after smoking cessation may help to reduce the risk of TB. In addition, it may be more helpful to quit smoking as soon as possible in order to reduce the risk of TB. Furthermore, Healthcare providers can use this information to improve the smoking cessation programs and tuberculosis prevention strategies.

## Supporting information

S1 Table Characteristics of subjects who underwent their regular health examinations whithin 2 years among current smokers. (DOCX)

S2 Table Relationship between body mass index at baseline and weight changes according to changes in smoking status of subjects who underwent their regular health examinations within 2 years among current smokers. (DOCX)
