# Supplementary material for: Association of weight change following smoking cessation with the risk of tuberculosis development: A nationwide population-based cohort study
Source: PLoS One. 2022 Apr 7;17(4):e0266262. doi: 10.1371/journal.pone.0266262 (PMC8989195; doi:10.1371/journal.pone.0266262)
Supplement: S1 Table — (DOCX) [file pone.0266262.s001.docx]

S1 Table. Characteristics of subjects who underwent their regular health examinations among current smokers after 2 years

|  | Quit smoking | | | Continued smoking | | | *p* value |
| --- | --- | --- | --- | --- | --- | --- | --- |
|  | Weight loss  (n=14,389) | Weight maintenance  (n=141,366) | Weight gain  (n=59,724) | Weight loss  (n=121,116) | Weight maintenance  (n=986,765) | Weight gain  (n=209,890) |  |
| Age | 47.1±13.6 | 46.3±12.1 | 43.5±12.2 | 46±12.8 | 44.1±11.3 | 39.9±11.4 | <.0001 |
| Age, years (grade) |  |  |  |  |  |  | <.0001 |
| 29-39 | 958 (6.7) | 7,513 (5.3) | 5,607 (9.4) | 8,178 (6.8) | 63,908 (6.5) | 33,737 (16.1) |  |
| 40-64 | 7,628 (53.0) | 80,157 (56.7) | 36,299 (60.8) | 68,239 (56.3) | 625,813 (63.4) | 135,610 (64.6) |  |
| ≥65 | 5,803 (40.3) | 53,696 (38.0) | 17,818 (29.8) | 44,699 (36.9) | 297,044 (30.1) | 40,543 (19.32) |  |
| Sex |  |  |  |  |  |  | <.0001 |
| Male | 13,294 (92.4) | 135,704 (96.0) | 56,895 (95.3) | 114,487 (94.5) | 958,262 (97.1) | 201,308 (95.9) |  |
| Female | 1,095 (7.6) | 5,662 (4.0) | 2,829 (4.7) | 6,629 (5.5) | 28,503 (2.9) | 8,582 (4.1) |  |
| Height, cm | 169.3±7.2 | 170.3±6.6 | 170.8±6.7 | 169.5±7.1 | 170.6±6.6 | 171.4±6.8 | <.0001 |
| Weight, kg | 66.6±11.0 | 71.1±10.5 | 73.2±11.0 | 65.9±11.1 | 70.2±11.0 | 73.4±11.9 | <.0001 |
| Waist circumference, cm | 81.2±8.0 | 84.2±7.8 | 85.3±7.8 | 80.8±8.0 | 83.1±8.0 | 84.6±8.3 | <.0001 |
| BMI, kg/m^2^ | 23.1±3.0 | 24.5±3.0 | 25.0±3.1 | 22.9±3.1 | 24.1±3.1 | 25.0±3.4 | <.0001 |
| BMI, kg/m^2^ (grade) |  |  |  |  |  |  | <.0001 |
| <18.5 | 796 (5.5) | 2355 (1.7) | 402 (0.7) | 8,082 (6.7) | 26,543 (2.7) | 2,115 (1.0) |  |
| 18.5-23 | 6,271 (43.6) | 41,507 (29.4 | 15,086 (25.3) | 56,500 (46.7) | 348,225 (35.3) | 59,913 (28.5) |  |
| 23-25 | 3,756 (26.1) | 39,441 (27.9) | 15,382 (25.8) | 28,850 (23.8) | 255,640 (25.9) | 50,577 (24.1) |  |
| 25-30 | 3,286 (22.8) | 52,507 (37.1) | 25,206 (42.2) | 25,268 (20.9) | 317,397 (32.2) | 81,499 (38.8) |  |
| ≥30 | 280 (2.0) | 5,556 (3.9) | 3,648 (6.1) | 2,416 (2.0) | 38,960 (4.0) | 15,786 (7.5) |  |
| Systolic blood pressure, mmHg | 122.2±14.1 | 124.1±13.5 | 124.9±13.3 | 122.7±13.9 | 123.6±13.4 | 124.0±13.1 | <.0001 |
| Diastolic blood pressure, mmHg | 76.4±9.7 | 77.9±9.5 | 78.4±9.4 | 76.9±9.6 | 77.7±9.5 | 77.9±9.3 | <.0001 |
| Glucose, mg/dL | 102.4±34.3 | 100.0±24.3 | 97.9±19.5 | 102.0±34.1 | 98.4±23.2 | 96.1±19.1 | <.0001 |
| Hemoglobin, g/dL | 14.6±1.4 | 14.9±1.2 | 14.9±1.2 | 14.9±1.3 | 15.2±1.2 | 15.2±1.2 | <.0001 |
| Cholesterol, mg/dL | 188.7±36.6 | 197.2±36.2 | 201.3±36.6 | 189.9±35.8 | 195.8±35.5 | 197.5±35.8 | <.0001 |
| Triglyceride ^a^, mg/dL | 112.5  (111.5-113.5) | 135.3  (134.9-135.7) | 145.5  (144.8-146.2) | 122.6  (122.2-123.0) | 141.4  (141.2-141.6 | 147.8  (147.4-148.2) | <.0001 |
| Place |  |  |  |  |  |  | <.0001 |
| Urban | 6,277 (43.7) | 62,288 (44.1) | 25,294 (42.4) | 51,739 (42.8) | 427,853 (43.4) | 88,007 (42.0) |  |
| Rural | 8,099 (56.3) | 79,006 (55.9) | 34,396 (57.6) | 69,225 (57.2) | 558,362 (56.6) | 121,734 (58.0) |  |
| Heavy drinker ^b^ |  |  |  |  |  |  | <.0001 |
| No | 12,502 (86.9) | 125,409 (88.7) | 52,283 (87.5) | 104,314 (86.1) | 846,258 (85.8) | 181,153 (86.3) |  |
| Yes | 1,887 (13.1) | 15,957 (11.3) | 7,441 (12.5) | 16,802 (13.9) | 140,507 (14.2) | 28,737 (13.7) |  |
| Regular exercise ^c^ |  |  |  |  |  |  | <.0001 |
| No | 4,862 (33.8) | 45,501 (32.2) | 21,383 (35.8) | 51,479 (42.5) | 389,302 (39.5) | 85,371 (40.7) |  |
| Yes | 9,527 (66.2) | 95,865 (67.8) | 38,341 (64.2) | 69,637 (57.5) | 597,463 (60.6) | 124,519 (59.3) |  |
| Diabetes | 2,171 (15.1) | 14,860 (10.5) | 4,343 (7.3) | 16,532 (13.7) | 87,957 (8.9) | 12,782 (6.1) | <.0001 |
| Hypertension | 3,883 (27.0) | 37,089 (26.2) | 14,220 (23.8) | 28,958 (23.9) | 216,330 (21.9) | 39,272 (18.7) | <.0001 |
| Dyslipidemia | 2,787 (19.4) | 30,110 (21.3) | 12,960 (21.7) | 19,648 (16.2) | 169,581 (17.2) | 34,128 (16.3) | <.0001 |
| Cerebrovascular disease | 250 (2.4) | 1,188 (1.1) | 433 (1) | 725 (0.9) | 3,454 (0.5) | 596(0.4) | <.0001 |
| Heart disease | 424 (4.0) | 3,112 (3.0) | 1,052 (2.4) | 1,610 (1.9) | 9,118 (1.3) | 1,469 (1.0) | <.0001 |
| Cancer | 911 (6.3) | 2,684 (1.9) | 950 (1.6) | 1,430 (1.2) | 6,036 (0.6) | 1,034 (0.5) | <.0001 |

BMI, body mass index. Values are number (%) or mean ± standard deviation. ^a^ Geographic means. ^b^ Defined as a person who drinks more than 30 gram of alcohol a day on average. ^c^ Defined as high-density exercise on more than 3 days a week for at least 20 minutes at a time or moderate-intensity exercise on more than 5 days a week for at least 30 minutes at a time
