# Supplementary material for: Association of weight change following smoking cessation with the risk of tuberculosis development: A nationwide population-based cohort study
Source: PLoS One. 2022 Apr 7;17(4):e0266262. doi: 10.1371/journal.pone.0266262 (PMC8989195; doi:10.1371/journal.pone.0266262)
Supplement: S2 Table — (DOCX) [file pone.0266262.s002.docx]

S2 Table. Relationship between body mass index at baseline and weight changes according to changes in smoking status of subjects who underwent their regular health examinations within 2 years among current smokers

|  | Bod mass index | | | | p-value |
| --- | --- | --- | --- | --- | --- |
|  | Underweight | Normal | Overweight | Obese |  |
| Continued smoking |  |  |  |  |  |
| Weight loss | 1915(4.88) | 35060(7.18) | 29180(8.75) | 54961(12.02) | <.0001 |
| Weight maintenance | 26918(68.65) | 354017(72.53) | 255383(76.62) | 350447(76.65) |  |
| Weight gain | 10377(26.47) | 99006(20.28) | 48734(14.62) | 51773(11.32) |  |
| Quit smoking |  |  |  |  |  |
| Weight loss | 165(3.12) | 3411(4.47) | 3485(6.02) | 7328(9.64) | <.0001 |
| Weight maintenance | 2636(49.82) | 44565(58.42) | 39506(68.26) | 54659(71.9) |  |
| Weight gain | 2490(47.06) | 28308(37.11) | 14888(25.72) | 14038(18.46) |  |

Values are number (%).
